# Sustainable Low-Cost Phosphorus Recovery Using Nanostructured Materials with Reusability Potential

**DOI:** 10.3390/nano13071167

**Published:** 2023-03-24

**Authors:** David Gómez-Carnota, José L. Barriada, Pilar Rodríguez-Barro, Manuel E. Sastre de Vicente, Roberto Herrero

**Affiliations:** Departamento de Química and CICA—Centro Interdisciplinar de Química e Bioloxía, Universidade da Coruña, As Carballeiras, s/n, 15071 A Coruña, Spain; david.gcarnota@udc.es (D.G.-C.); pilar.rbarro@udc.es (P.R.-B.); manuel.sastre@udc.es (M.E.S.d.V.); r.herrero@udc.es (R.H.)

**Keywords:** phosphorus, adsorption, nanostructures, recovery, iron, environmental remediation

## Abstract

A new low-cost material with a polymeric base formed from sodium silicate was developed. The material presents a nanostructured, highly rich iron surface with a large phosphorus retention capacity and potential reuse as a crop fertilizer. In the present study, we demonstrate that iron is the element that acts as an adsorbent for phosphate, while the polymeric base functions exclusively as a support for iron. The iron is uniformly adsorbed on the surface of the material, forming nanostructures, which ensure that iron works similarly to nanoparticles in solution but avoid other problems, such as particle agglomeration or the difficulty of separating them after the removal process. Materials were characterised by SEM, EDS, N_2_ sorption, and image processing, and the effect of pH, ionic strength, and temperature was studied. Sorption kinetics were analysed using Boyd’s diffusion model, and adsorption equilibria were studied using several adsorption models. A maximum iron adsorption on the polymeric base of 23.9 ± 0.3 mg Fe∙g^−1^ was found, while maximum phosphorus adsorption was 366 ± 21 mg P∙g^−1^ Fe. Thus, phosphorus is recovered from the aqueous medium with an inexpensive material that has the potential to be used directly as a fertilizer.

## 1. Introduction

Raw materials are becoming increasingly expensive to obtain from nature due to their scarcity. In 2019, in the context of the international year of the periodic table of the chemical elements, the European Chemical Society presented an innovative design of the periodic table. It illustrates the current availability of the elements in nature. Many elements that are vital to society are under serious threat of depletion, there is limited availability with risk to supply in the next few years, or they are found in conflict minerals [1]. For these reasons, it is necessary to evolve towards waste treatment systems that make it possible to recover these elements and reuse them as raw materials, establishing circular economy systems.

This work applies these principles to phosphate removal or recovery. Phosphorus is an element classified as being of limited availability with future risk to supply [1]. As a raw material, it is obtained as phosphate from several minerals. The most abundant ones are forms of apatite, Ca_5_(PO_4_)_3_X (being X fluoride, chloride, or hydroxyl). Most of the extracted ore is transformed into phosphoric acid (H_3_PO_4_). About 50 million tonnes of phosphoric acid are produced each year, which is mainly used to produce phosphate fertilisers. Phosphates were also widely used in detergents from 1945 until a few years ago. The decline in their use is strongly linked to the high emissions of phosphates to water originating from their use. Other examples of industrial uses of phosphates are glass and ceramic fabrication, leather tanning, and toothpaste production [2]. The European Union lists phosphates and organophosphorus compounds as priority pollutants in legislation for industrial emissions and in water policy [3,4]. Environmental agencies also warn of the problems caused by these pollutants. For example, the United States Environmental Protection Agency recognises nutrient pollution caused by excess phosphates and nitrates in water bodies as one of the most costly, widespread, and challenging environmental problems in the United States [5]. The excessive presence of nutrients in water causes an accelerated eutrophication of aquatic systems. This consists of massive growth of algae or plants, causing undesirable changes in water quality and ecological imbalances. The main problems of eutrophication are overgrowth of species whose blooms can be toxic, excessive oxygen consumption leading to the death of aerobic aquatic organisms, and the spread of decomposer bacteria due to high mortality [6].

In Europe, under the Industrial Emissions Directive 2010/75/EU [7], a dataset containing the location and administrative data for the largest industrial complexes in Europe has been available since 2007. Over 550,000 tonnes of phosphorus were emitted to waters in the period 2007–2020, of which more than 16,000 tonnes were emitted in 2020 (several countries did not report their emissions in the period 2017–2020) [8]. These data correspond only to the data of the largest industrial complexes. However, the use of phosphate fertilisers is widespread. This can result in much larger emissions of phosphates to water through surface run-off into rivers and lakes or leaching into groundwater [9,10,11].

Many countries have problems with the pollution of rivers and lakes due to phosphate emissions. The European Environment Agency has monitored several rivers and lakes in European countries since 2016. Countries such as France, Ireland and Denmark are among those with the most problems with phosphorus pollution in lakes [12]. Phosphate pollution in rivers is much worse, affecting a large number of European countries, such as Spain, Italy, the UK, Denmark, Belgium, and Poland, among others [13].

Phosphorus is a pollutant emitted by numerous industries, manufacturing, agriculture, and livestock. Examples are swine farming ([P] 100–900 mg/L, pH 6–9 [14,15,16]), slaughterhouses, ([P] 25–200 mg/L, pH 4.9–8.1 [17]), phosphoric acid manufacture ([P] 15–31 mg/L, pH 2.7–2.8 [18]), phosphogypsum lixiviation ([P] 4415–5832 mg/L, pH 2.2–2.5 [19]) or anodising industry ([P] 202–1653 mg/L, pH 1.9–6.6 [20]).

Different techniques have been tried to remove this pollutant from industrial effluents to prevent the problems of phosphorus emissions into the environment. Some of these techniques are precipitation, membrane purification, coagulation and flocculation, ion exchange, biological uptake, or adsorption [6,21,22].

Adsorption is a technique that offers great advantages for the reuse of phosphorus once it has been removed from the polluted effluent. This technique allows the phosphorus to be immobilised in a material specifically designed to be used later as a raw material. Phosphorus can be recovered in two ways using this technique: phosphate elution or direct use of the material as fertiliser [14]. In this work, a phosphate removal material has been developed based on the following strategy.

- The material has been synthesised from cheap and abundant raw materials (silicate and iron) using a process that is as simple as possible to keep costs as low as possible.
- The material has been designed with the reuse of the pollutant in mind. With a low-cost material design and components after phosphorous addition that allow the material to be reused directly, we will achieve a waste adsorbent with enormous added value.

As a result of this strategy, in this work we developed a polymeric base from sodium silicate and water, which we functionalised with iron by adsorption.

Polymeric materials are a good choice when designing an adsorbent material. This type of material is usually porous, which allows good diffusion of the pollutant through the surface of the material, facilitating its adsorption. Functionalisation of these polymeric materials using different types of ions or compounds also tends to increase the adsorption capacity of the material if an ion or compound related to the pollutant is used. Examples of this type of material development technique are zeolites incorporating ions in their cavities, hydrogels with metals in their structure, and biochar impregnated with different compounds [14].

Although functionalisation of materials is advantageous to maximise adsorption of a contaminant, it becomes a major problem when low-abundance ions are used for functionalisation or the material requires many or complex steps for its synthesis [23,24,25].

Our material design attempts to solve these problems by using low-cost materials with as simple and as few steps as possible for their synthesis.

## 2. Materials and Methods

### 2.1. Chemicals and Measurement Methods

Sodium silicate neutral solution, EssentQ, from Scharlau (Scharlab S.L., Barcelona, Spain). HCl 37% PA-ACS-ISO from Panreac (Panreac Química S.A., Barcelona, Spain) and iron sulphate 7-hydrate PA-ACS were used in the synthesis of the materials of this study. 1,10-phenantroline 1-hydrate PA-ACS, acetic acid glacial purissimum and sodium acetate 3-hydrate (RFE, USP, BP Ph. Eur.) were used for Fe(II) measurements. The phenanthroline UV-vis standard method was used for the Fe(II) measurements (Zuzi spectrophotometer model 4211/20, AUXILAB, S.L., Beriáin, Spain). In brief, 1,10-phenanthroline forms an orange-red complex with Fe(II) ions in a buffered medium. To determine the Fe(II) concentration, the absorbance of the complex at 510 nm is measured [26]. Potassium dihydrogen phosphate p.a. ISO, potassium antimony (III) tartrate 3-hydrate PA-ACS, ammonium molybdate 4-hydrate (Reag. Ph. Eur.) PA-ACS-ISO and L(+)-ascorbic acid PA-ACS from Panreac were used for phosphorus measurements. The ascorbic acid UV-vis standard method was used for the phosphorus measurements. In brief, ammonium molybdate, antimony tartrate and ascorbic acid are used to synthesise a combined reagent that reacts with phosphate ions to form molybdenum blue, which is deep blue. To determine the phosphate concentration, the absorbance of this compound is measured at 880 nm [27]. HCl 37% PA-ACS-ISO, NaOH 98% ACS-ISO and from Panreac were used for pH adjustment. All solutions were made with deionised water.

### 2.2. Synthesis of Materials

Two different materials were synthesised in this work. To synthetise the first one (named granulated silica pellets, code GSP), 30 mL of a 1.8 M Na_2_SiO_3_ solution was mixed with 30 mL of deionised water. After homogenisation, 3.6 mL of 4 M HCl was added. The mixture was stirred until polymerisation began. The formed gel was poured into a homemade casting mould with holes of 7 mm in diameter and 5 mm in height. The mould was placed in an air turbine oven (Memmert beschickung-loading model 100–800, Memmert GmbH, Schwabach, Germany) for 24 h at 25 °C to dry the gel. After drying, the material obtained (code GSP) was crushed and sieved. Three particle sizes were separated for the experiments: GSP between 0.5 and 1 mm in size (code GSP S-1), GSP between 0.25 and 0.5 mm in size (code GSP S-0.5) and GSP below 0.25 mm in size (code GSP S-0.25).

The second material (code GSP-Fe) was synthetised by submerging 0.2 g of GSP of the three selected particle sizes into 100 mL solutions with a Fe(II) concentration of 250 mg Fe(II)∙L^−1^. Solutions were stirred at 175 rpm, room temperature and natural pH for 24 h. Finally, the synthesised GSP-Fe was dried in an air turbine oven at 25 °C. Codes GSP-Fe S-1, GSP-Fe S-0.5 and GSP-Fe S-0.25 were assigned in an analogous way to the codes assigned to the different GSP sizes.

### 2.3. Characterisation

N_2_ physisorption analyses (Tristar II Plus 3030 surface area and porosity analyser, Micrometrics Instruments Corporation, Norcross, GA, USA) were performed for the three particle sizes used in this work to determine the surface and porosity of GSP, GSP-Fe and GSP-Fe after phosphorus sorption. The surface and composition of the materials were studied by scanning electron microscopy (JSM-7200F, Jeol Ltd., Tokyo, Japan) and energy-dispersive X-ray spectroscopy (Oxford EDS X-Max^N^ detector, Oxford Instruments, Abingdon, England) analyses.

### 2.4. Effect of Solution pH

GSP-Fe S-1 (0.1 g) was added to 50 mL solutions with phosphorus concentration of 25 mg∙L^−1^. pH was modified by adding HCl or NaOH from pH 1 to pH 7.5. Solutions were stirred for 24 h at room temperature.

### 2.5. Kinetic Studies

Iron sorption kinetics were studied by adding 0.2 g of GSP of GSP S-1 and S-0.5 to 100 mL solutions with an iron concentration of 80 mg Fe(II)∙L^−1^. Solutions were stirred at 175 rpm, room temperature, and natural pH.

To check the influence of Fe(II) initial concentration on the kinetics, 0.2 g of GSP S-1 was added to 100 mL solutions with different Fe(II) concentrations (from 50 to 250 mg Fe(II)∙L^−1^) in 250 mL Erlenmeyer flasks. Solutions were stirred at 175 rpm, room temperature and natural pH.

Phosphorus sorption kinetics were studied by submerging 0.2 g of GSP-Fe S-1 and S-0.5 in 100 mL solutions with a phosphorus concentration of 33 mg∙L^−1^. Solutions were stirred for 24 h at 175 rpm, room temperature and pH 2.1.

### 2.6. Equilibrium Studies

Iron studies were performed by adding 0.1 g of GSP S-1, S-0.5 and S-0.25 to 50 mL of different Fe(II) solutions with concentrations between 30 and 110 mg Fe(II)∙L^−1^. Solutions were stirred for 24 h at 175 rpm, room temperature and natural pH.

The dependence of phosphorus adsorption on the iron adsorbed on GSP-Fe was also studied. GSP S-1 (0.2 g) was immersed in Fe(II) solutions with concentrations between 10 and 50 mg Fe(II)∙L^−1^ and with the conditions described in Section 2.2. After iron uptake, the resulting materials were immersed in 50 mL solutions with a phosphorus concentration of 50 mg∙L^−1^. The solutions were stirred for 24 h at 175 rpm, room temperature and pH 2.1.

Phosphorus sorption studies were done by adding 0.1 g of GSP-Fe S-1, S-0.5 and S-0.25 to 50 mL of several phosphorus solutions with concentrations between 3 and 130 mg∙L^−1^. Solutions were stirred for 24 h at 175 rpm, room temperature and pH 2.1.

Finally, the effect of ionic strength and temperature was studied. of GSP-Fe S-1 (0.1 g) was added to several 50 mL of 80 mg∙L^−1^ phosphorus solutions with ionic strength from 0.05 M to 0.5 M. Ionic strength was adjusted adding KNO_3_. Temperature effects were studied with GSP-Fe S-1 and S-0.25, with 0.1 g added to several phosphorus solutions with concentrations of 3–150 mg∙L^−1^ at different temperatures (from 8 °C to 52 °C).

## 3. Results and Discussion

### 3.1. Characterisation of the Materials

**Figure 1:**
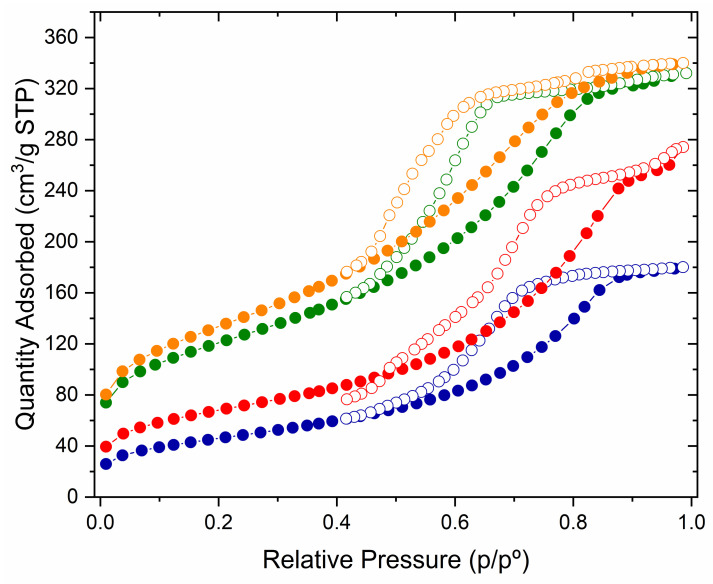
N_2_ adsorption (filled circles) and desorption (empty circles) isotherm of GSP S-1 (blue), GSP S-1 washed with deionised water to remove NaCl deposits (red), GSP-Fe S-1 (green), and GSP-Fe S-1 with adsorbed phosphorus (orange). T = 77 K.

GSP is a semi-translucent material with small white powdery deposits. SEM and EDS analysis revealed that this material is composed by a solid structure of silicon and oxygen ions. NaCl deposits appear on the surface from the reaction between Na_2_SiO_3_ and HCl. N_2_ physisorption analyses were performed for the three particle sizes used in this work to determine the surface area and porosity of the synthesised GSP. Experimental data from all N_2_ adsorption experiments performed conform to a type IV isotherm among those described by Brunauer et al. [28,29] (Figure 1).

The parameters obtained from the experiments are summarised in Table 1. As can be seen, the BET surface area decreases with decreasing particle size. This is a strange behaviour, because the surface area of a material usually increases as its particle size decreases. In this case, the decrease in surface area is linked to the presence of NaCl compact deposits on the surface of the material and to the porosity reduction of the material when the particle size decreases. As the size and pore volume of the material decreases, the surface area on which N_2_ can be adsorbed decreases.

This effect was evaluated by washing the GSP with deionised water. This washing dissolves the compact NaCl deposits present on the GSP and exposes the porous surface formed by silicon and oxygen. As can be seen in Table 1, as the surface of the GSP is exposed after washing, the porosity and the BET surface increase for the three particle sizes. In addition, after this washing, the surface area increases as the particle size decreases, which is the usual behaviour of the materials.

Figures showing experimental data of N_2_ sorption, pore size distribution and the cumulative pore volume of fraction S-1 tests can be found in the Supplementary Materials.

**Figure 2:**
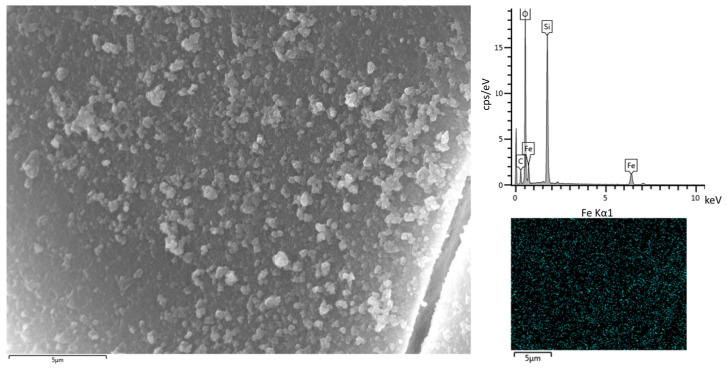
SEM image (5000× magnification), EDS spectrum and EDS map (Fe, cyan) of GSP-Fe S-1.

GSP is a material that does not adsorb phosphorus. This will be discussed in more detail in Section 3.4.2. Because of this fact, GSP is transformed by Fe(II) adsorption into a new material. This material, called GSP-Fe, has a characteristic reddish-brown colour. SEM and EDS analysis revealed that iron-rich nanostructures appear on the surface of the GSP-Fe, which are not present in GSP surface. This indicates that the nanostructures are formed during the iron adsorption process on the GSP surface. Figure 2 shows the surface of GSP-Fe S1 as an example. No significant differences in the surface of GSP-Fe among the three fractions of the material (S-1, S-0.5 and S-0.25) were observed. Figure 2 also shows that NaCl is not detected in the material, which means that it was completely washed away in the Fe(II) solution used for the synthesis.

N_2_ sorption tests were also conducted on GSP-Fe.

The formation of the iron-rich nanostructures further increases the BET surface respect to the washed GSP, for the tree particle sizes (Table 1). Moreover, as the nanostructures are formed over the porous silicate surface, the porosity of the GSP-Fe is lower than the porosity of the washed GSP.

**Figure 3:**
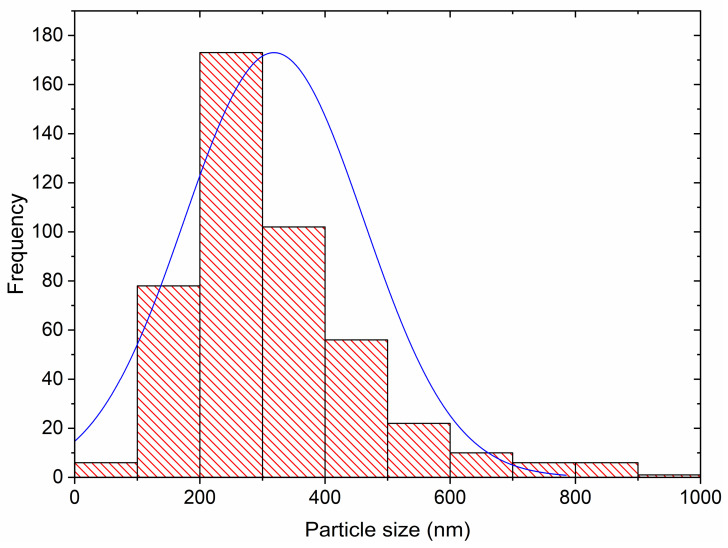
Histogram of the size of the iron-rich nanostructures present on the GSP-Fe surface. ▬ Normal distribution curve.

The surface of the GSP-Fe was studied to determine the size of the iron-rich nanostructures. SEM images were analysed with an image processing and analysis programme called ImageJ [30]. The histogram resulting from these analyses is shown in Figure 3. The average particle diameter is 318 nm, with a SD of 143 nm. Most of the particle diameters are in the range 200–300 nm and the normal distribution curve indicates that 99% of the particle diameters are between 0 and 747 nm.

The oxidation state of the iron adsorbed on the GSP-Fe was determined by XPS analysis [31]. The main oxidation state is Fe(III) (66.12%), and the rest is Fe(II) (34.18%). However, this material was prepared using an Fe(II) solution, so when the GSP is immersed in the solution, very fast oxidation of Fe(II) to Fe(III) occurs on the surface of the GSP after its sorption. This fact was confirmed by an Fe(II) adsorption experiment in an inert atmosphere. As long as the inert atmosphere was maintained, there was adsorption of Fe(II) on GSP, but no oxidation. This could be verified because the colour of the GSP-Fe under inert atmosphere was blue rather than red. When the inert atmosphere was removed, the adsorbed Fe(II) began to oxidise to Fe(III), until the colour changed completely to the reddish-brown characteristic of GSP-Fe.

Further information such as pore size distribution (hollow diamonds) and cumulative pore volume of GSP-S1 materials (Figure S1), a SEM image, EDS spectra, EDS maps of GSP S-1 (Figures S2 and S3), and a SEM image and EDS maps of GSP-Fe S-0.25 (Figure S4) can be found in the Supplementary Materials.

### 3.2. Effect of Solution pH

Previous studies of iron adsorption using GSP in pellet form revealed that the optimum pH for adsorption is the natural pH of the solution (approximately 5) [31]. At more acidic pH, adsorption decreases due to the protonation of the silicate oxygens. When the pH is increased by adding a base, Fe(II) is oxidised to Fe(III) and the solution becomes cloudy. This is a sign that the iron present in the solution precipitates. When this effect was observed, it was also found that iron adsorption on GSP decreased.

**Figure 4:**
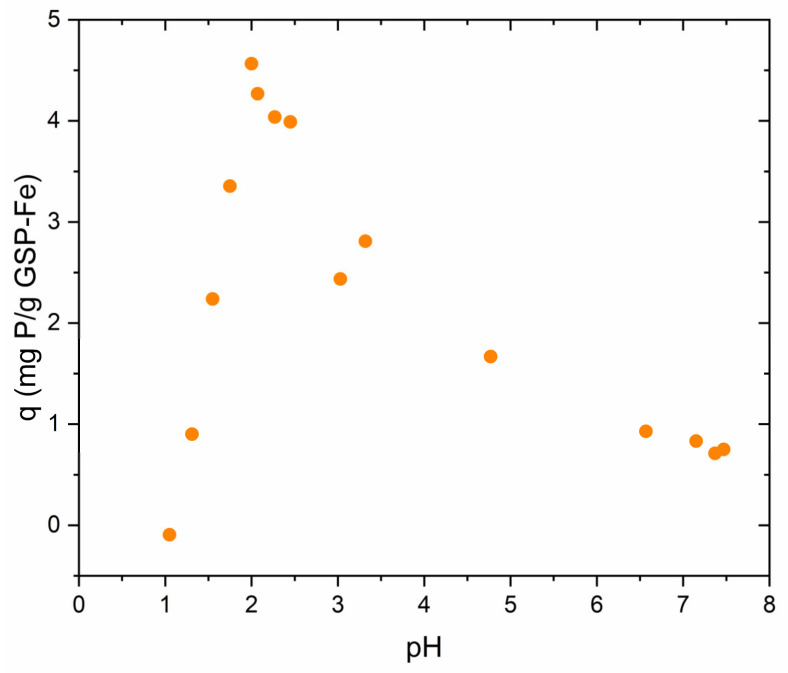
Effect of solution pH on phosphorus adsorption using GSP-Fe S-1. Dose of GSP-Fe S-1 = 2 mg∙L^−1^, equal to 47.4 mg∙L^−1^ of Fe, room temperature and stirring at 175 rpm.

The optimum pH for phosphorus adsorption on GSP-Fe was also studied. Experimental data are shown in Figure 4. Adsorption is maximised when the pH of the medium is about 2.1. From these conditions, a gradual decrease in adsorption is observed as the solution pH increases. When the pH is below 2, phosphorus adsorption decreases sharply with decreasing pH. This effect occurs because at pH below 2, the iron present in the GSP-Fe starts to desorb. The effect is faster the closer the pH is to 1.

### 3.3. Adsorption Kinetics

**Figure 5:**
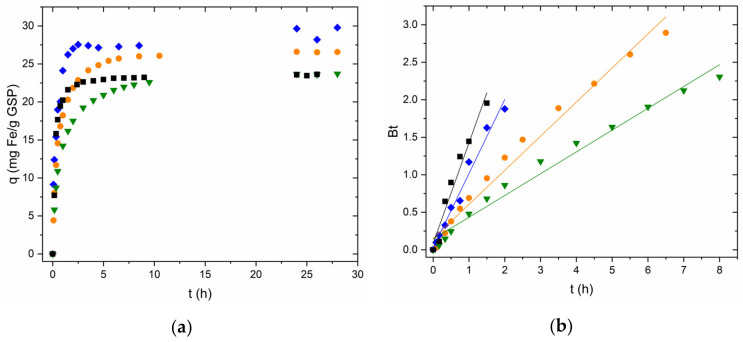
(**a**) Fe(II) adsorption kinetics using GSP S-1 (initial Fe(II) concentration: ▼ 50 mg∙L^−1^; ● 80 mg∙L^−1^; ♦ 275 mg∙L^−1^) and S-0.5 (■, initial Fe(II) concentration 80 mg∙L^−1^). (**b**) Application of Boyd model to the Fe(II) adsorption kinetics. Natural pH, room temperature and stirring at 175 rpm for all experiments.

Reaction kinetics were analysed using the lineal Boyd diffusion model [32,33]. Experiments were performed using the S-1 and S-0.5 fractions of GSP and GSP-Fe. Figure 5a shows different adsorption kinetics of Fe(II) on GSP. It can be seen that for the same particle size, the reaction rate is accelerated when the initial Fe(II) concentration in solution is larger. It is also observed that for the same initial concentration, the reaction is faster as the particle size decreases.

Figure 5b shows how the linearised Boyd model correctly describes the experimental data. Fitting lines with good Pearson’s r values (0.980–0.991) are obtained. As shown in Table 2, for the two fastest kinetics, considering the error, the fit goes through zero. This indicates that these two reactions are compatible with a process governed by intraparticle diffusion. In the case of the two slower reactions, the error does not contain the ordinate. Therefore, these two reactions are compatible with a process governed by film diffusion [34,35].

To corroborate these hypotheses, the intercepts were analysed by applying Student’s *t*-test at 95% confidence. For the two fastest kinetics, the intercept is statistically 0. Following this approach, the adsorption process of Fe(II) in these reactions is a diffusion process in which the limiting step is intraparticle diffusion. In the case of the slower kinetics, the ordinate is not statistically 0, so the limiting step would be film diffusion. All *t*-tests agree with the data obtained from the errors of the ordinates.

**Figure 6:**
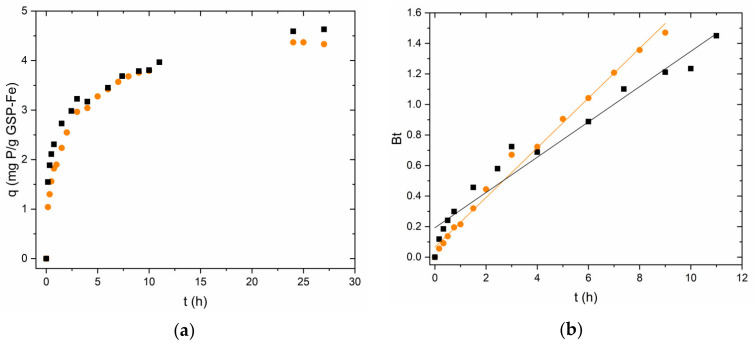
(**a**) Phosphorus adsorption kinetics using GSP-Fe S-1 (●, initial P concentration 33 mg∙L^−1^) and S-0.5 (■, initial P concentration 33 mg∙L^−1^). (**b**) Application of Boyd model to the phosphorus adsorption kinetics data. pH 2.1, room temperature and stirring at 175 rpm for all experiments.

The adsorption rate of phosphorus on GSP-Fe was studied using the same particle sizes as in the iron studies (S-1 and S-0.5). Figure 6a shows that in this case, the particle size has no clear influence on the adsorption rate. In this case, the linearised Boyd model also describes properly the experimental data (Pearson’s r: 0.979–0.996, Figure 6b, Table 2). This indicates that the phosphorus adsorption process on GSP-Fe is also a diffusion process. In both cases, the intercept is not statistically 0, so according to this approach, the limiting step is the film diffusion.

### 3.4. Equilibrium Studies

#### 3.4.1. Iron Equilibrium Studies

**Figure 7:**
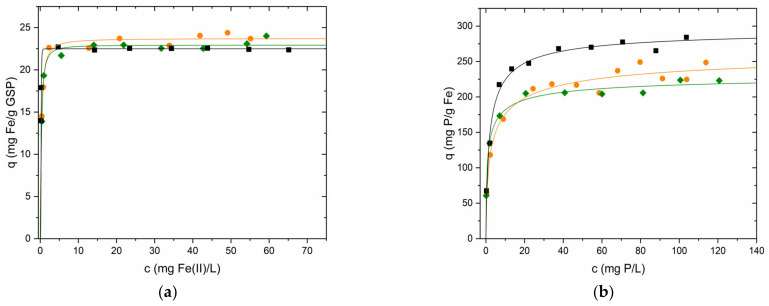
(**a**) Experimental data of Fe sorption on GSP (●) S-1, (♦) S-0.5, (■) S-0.25; solid lines were obtained using the Langmuir–Freundlich model (Equation (S6)). GSP dose 2 g∙L^−1^, natural pH, and room temperature. (**b**) Experimental data of phosphorus sorption on GSP-Fe referring to the iron. Solid lines were obtained using Equation (S6). (●) GSP-Fe S-1. Dose 2 g∙L^−1^, which is equivalent to 47.4 mg Fe∙L^−1^. (♦) GSP-Fe S-0.5. Dose 2 g∙L^−1^, equivalent to 45.8 mg Fe∙L^−1^. (■) GSP-Fe S-0.25. Dose 2 g∙L^−1^, equivalent to 45 mg Fe∙L^−1^. pH 2.1 and room temperature for all phosphorus experiments.

The adsorption equilibria of Fe(II) on GSP were studied using the three synthesised particle sizes (S-1, S-0.5 and S-0.25). Experimental data are shown in Figure 7. Solid lines were obtained by fitting to the Langmuir–Freundlich model. This model and the model described by Sellaoui et al. [36] (Equation (S7)) are those that best describe the experimental data (R^2^: 0.958–0.998). The parameters obtained by applying the models are summarised in Table 3. As can be seen, regardless of its size, GSP has a very high affinity for Fe(II). This fact is represented by the almost vertical slope of the isotherm before saturation. In addition, the high adsorption energies calculated using Equation (S8) [37] (34.3–34.9 kJ∙mol^−1^) are consistent with the high affinity of GSP for iron. The Q_0_ values obtained by the 3 models are remarkably similar for each particle size. In the case of the Langmuir–Freundlich and Sellaoui models, the value of Q_0_ is the same, while the values of K and n are the reciprocals of c_½_ and m, respectively. They are also consistent with the surface area values of the three GSP sizes seen in Section 3.1 (Table 1). Among the three particle sizes, the values of Q_0_ are also remarkably similar (22.5–23.7 mg Fe∙g^−1^ GSP).

Extended information such as the experimental data fitted with the Langmuir model (Equation (S5) (Figure S5a)) and the Sellaoui et al. model (Equation (S7) (Figure S6a)) can be found in the Supplementary Materials.

#### 3.4.2. Correlation between Phosphorus Adsorption and Iron Present in GSP-Fe

Two experiments were conducted to prove there is no phosphorus adsorption on GSP, and that this adsorption is linked to the iron present in the GSP-Fe. In the first experiment, the adsorption of phosphorus on GSP at different pH was analysed. As a result, it was found that there is no adsorption in the pH range studied (1–8).

In the second experiment, several fractions of GSP S-1 were functionalised with solutions of different Fe concentrations (from 10 to 50 mg Fe(II)∙L^−1^). After the iron adsorption was completed, the material was used to adsorb phosphorus. There is no phosphorus adsorption on GSP-Fe until the material has a minimum of 0.0435 millimoles of iron. Thereafter, the correlation between phosphorus adsorption and iron present in the material is linear ($q_{P}=(740\;\pm \;6)\cdot 10^{-3}q_{\mathrm{Fe}\;}-\;(321\;\pm \;5)\cdot 10^{-4}$. Phosphorus sorption is increased by 0.7395 millimoles per millimole of iron present in the material, until the GSP is saturated with iron.

A figure showing the data from the experiments of this section can be found in the (Supplementary Materials Figure S7).

#### 3.4.3. Phosphorus Equilibrium Studies

Adsorption equilibrium experiments at room temperature using the three synthesised sizes of GSP-Fe (S-1, S-0.5 and S-0.25) were conducted. The experimental data are shown in Figure 7. The solid lines were obtained using the Langmuir–Freundlich model (Equation (S6)) because it is again the model that best describes the experimental data, together with the Sellaoui et al. model. (R^2^ 0.958–0.992). The parameter values obtained with the three models and their errors are listed in Table 3. As can be seen, there are certain differences in the values of Q_0_ in the fractions S-1 and S-0.25 due to the shape of the isotherms near saturation. In this case, the errors in the parameters Q_0_, K and c½ are higher in the experiments using GSP-Fe S-1 since the saturation region of the isotherm is not as well defined as in the experiments with S-0.5 and S-0.25. The error of n and m is smaller because the slope of the isotherm is better defined. The adsorption energy of phosphorus on GSP-Fe is also high, being similar to the adsorption energy of iron on GSP.

The maximum phosphorus adsorption values obtained with this material are good, in particular those obtained with the finest fraction of material (S-0.25). The maximum adsorption obtained with the largest fraction (S-1) is not far behind, being 16% lower, but in return the material is easier to handle. The intermediate fraction (S-0.5) is the worst performing of the three, being 22% lower than the S-0.25 fraction.

#### 3.4.4. Effect of Ionic Strength and Temperature on Phosphorus Sorption

The influences of ionic strength and temperature on the phosphorous adsorption were studied.

Starting with ionic strength studies, a small difference in phosphorus adsorption values at different ionic strengths was found (q = 224 ± 17, I = 0.05; q = 227 ± 3, I = 0.1; q = 225 ± 7, I = 0.2; q = 196 ± 11, I = 0.5). However, this variation is within the range of the experimental error. This is very positive for retaining phosphorus in different aqueous media. The results show that the material can be used in saline medium, as no competition or decrease in phosphorus adsorption capacity is observed.

**Figure 8:**
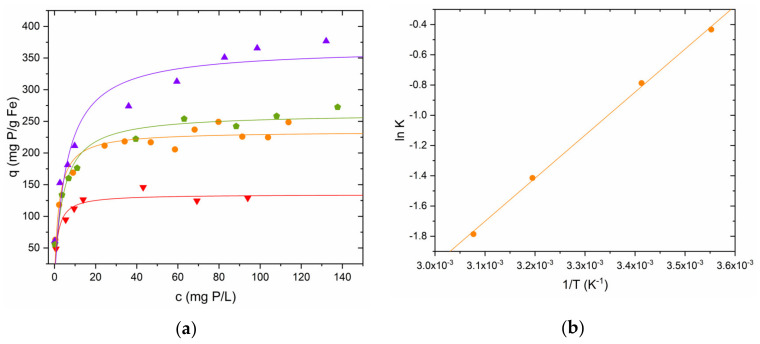
(**a**) Influence of temperature on phosphorus adsorption using GSP-Fe S-1. Dose 2 g∙L^−1^, equivalent to 47.4 mg Fe∙L^−1^. (▼) 8.5 °C, (●) 20 °C, (⭓) 40 °C, (▲) 52 °C. pH 2.1 for all experiments. (**b**) Representation of ln K vs 1/T according to van ’t Hoff equation.

The phosphorus adsorption equilibrium on GSP-Fe was studied at several temperatures. Two particle sizes were used to study this effect: the largest (S-1) and the smallest (S-0.25). Figure 8a shows the isotherms performed with GSP-Fe S-1 as an example. Temperature effect on maximum adsorption is observed, especially at low temperature. In this case, the Langmuir model is used to describe the experimental data, because it is the model that best fits them. It is also the model with the smallest errors of the parameter K. The values obtained from the fitting are summarised in Table 4.

The parameter K obtained from the Langmuir model was used to calculate the enthalpy of reaction using the van ’t Hoff equation. Figure 8b shows the representation of ln K vs. 1/T with the data obtained in the phosphorus adsorption using GSP-Fe S-1 experiments as example. The reaction enthalpies are also shown in Table 4. Both settings show the same behaviour: a straight line with a particularly good linear correlation coefficient (0.999 in both fits) and positive slope is obtained. The positive slope in this type of fit is characteristic of an exothermic reaction (ΔH° < 0).

When the temperature is increased up to 52 °C, we obtain an increase in maximum phosphorus adsorption of 56% in the S-1 fraction, and an increase of 29% in the S-0.25 fraction. At this temperature, the maximum adsorptions of both fractions are practically equal. This would make it possible to choose the fraction that is easiest to reuse for phosphorus adsorption.

Table 5 shows the performance of the material synthesised in this work, compared to other reports of phosphorus adsorption obtained from the literature.

Further information, such as the experimental equilibrium data fitted with the Langmuir model (Equation (S5) (Figure S5b)) and the Sellaoui et al., model (Equation (S7) (Figure S6b)), influence of the ionic strength (Figure S8), influence of the temperature using GSP-Fe S-0.25, and its representation of ln K vs. 1/T (Figure S9) can be found in the Supplementary Materials.

## 4. Conclusions

The material synthesised in this work, consisting of a sodium silicate polymeric base functionalised with iron (code GSP-Fe) is a material with iron-rich nanostructures on its surface (a maximum of 23.9 ± 0.3 mg Fe∙g^−1^ of polymeric base). This nanoscale iron can remove considerable amounts of phosphorus (up to 366 mg P∙g^−1^ Fe) while avoiding the typical problems of nanoparticles in solution.

Phosphorus adsorption does not decrease with increasing salinity of the aqueous medium when GSP-Fe is used as adsorbent, which is greatly advantageous in recovering phosphorus in a wide variety of aqueous environments.

As GSP-Fe after the decontamination process is a material composed of silicon, oxygen, iron, and phosphorus, it is potentially directly usable as crop fertiliser.

## Figures and Tables

**Table 1:** Surface parameters of GSP, GSP-Fe and GSP-Fe with adsorbed phosphorus.

Material	Particle Size	BET Surface (m^2^∙g^−1^)	Pore Volume (cm^3^∙g^−1^)	Average Pore Diameter (nm)
GSP	S-1	164.62	0.292	5.82
	S-0.5	158.29	0.265	5.60
	S-0.25	131.83	0.217	5.54
GSP washed with deionised water	S-1	239.51	0.436	5.92
	S-0.5	283.22	0.446	6.31
	S-0.25	287.87	0.501	6.74
GSP-Fe	S-1	422.12	0.457	4.97
	S-0.5	447.75	0.472	4.58
	S-0.25	464.92	0.477	5.03
GSP-Fe with P	S-1	470.86	0.450	4.60

**Table 2:** Kinetic parameters obtained using the Boyd diffusion model for Fe and P adsorption.

Reaction	Kinetic Parameters			Boyd Lineal Model			
	Particle Size	c_i_ (mg∙L^−1^)	pH	Intercept	Pearson’s r	Experimental Student’s t	Critical Student’s t (95%)
Fe(II) sorption on GSP	S-1	50	Nat.	0.14 ± 0.05	0.990	2.94	1.80
	S-1	80	Nat.	0.15 ± 0.05	0.991	2.88	1.78
	S-1	275	Nat.	0.03 ± 0.05	0.991	0.57	1.89
	S-0.5	80	Nat.	0.09 ± 0.09	0.980	0.92	2.02
P sorption on GSP-Fe	S-1	33	2.1	0.065 ± 0.018	0.996	3.72	1.77
	S-0.5	33	2.1	0.19 ± 0.04	0.979	4.97	1.78

**Table 3:** Sorption parameters obtained from the isotherm models for iron adsorption on GSP and phosphorus adsorption on GSP-Fe at room temperature (20 °C).

Isotherm Model	Particle Size	Langmuir Model			Langmuir-Freundlich Model				Sellaoui et al. Model				
		Q_o_ (mg∙g^−1^)	K (L∙mg^−1^)	R^2^	Q_o_ (mg∙g^−1^)	K (L∙mg^−1^)	n	R^2^	Q_o_ (mg∙g^−1^)	m	c_1/2_ (mg∙L^−1^)	R^2^	E (kJ∙mol^−1^)
Fe(II) sorption by GSP	S-1	23.9 ± 0.3	3.9 ± 0.4	0.959	23.7 ± 0.3	3.5 ± 0.5	0.83 ± 0.16	0.958	23.7 ± 0.3	1.2 ± 0.2	0.29 ± 0.04	0.958	34.5
	S-0.5	23.2 ± 0.3	4.0 ± 0.4	0.956	22.9 ± 0.3	3.3 ± 0.4	0.71 ± 0.14	0.960	22.9 ± 0.3	1.4 ± 0.3	0.31 ± 0.04	0.960	34.3
	S-0.25	22.7 ± 0.3	8.2 ± 1.2	0.919	22.49 ± 0.05	4.19 ± 0.04	0.200 ± 0.010	0.998	22.49 ± 0.05	5.0 ± 0.2	238∙10^−3^ ± 2∙10^−3^	0.998	34.9
Phosphorus sorption referred to the iron present on GSP-Fe	S-1	234 ± 6	0.46 ± 0.11	0.922	270 ± 24	0.29 ± 0.13	1.7 ± 0.4	0.958	270 ± 24	0.58 ± 0.12	3.4 ± 1.5	0.958	31.6
	S-0.5	212 ± 6	1.1 ± 0.3	0.920	234 ± 10	0.9 ± 0.2	1.8 ± 0.2	0.983	234 ± 9	0.56 ± 0.07	1.1 ± 0.3	0.983	34.5
	S-0.25	278 ± 6	0.52 ± 0.09	0.967	297 ± 11	0.48 ± 0.08	1.40 ± 0.16	0.983	297 ± 11	0.71 ± 0.08	2.1 ± 0.4	0.983	32.9

**Table 4:** Sorption parameters obtained from the Langmuir model for phosphorus adsorption on GSP-Fe at several temperatures.

Equilibrium	Particle Size	T (°C)	Langmuir Model			van ’t Hoff Equation	
			Q_o_ (mg∙g^−1^)	K (L∙mg^−1^)	R^2^	ΔH° (kJ∙mol^−1^)	Pearson’s r
Phosphorus sorption referred to the iron present on GSP-Fe	S-1	8	134 ± 6	0.65 ± 0.20	0.899	−23.7 ± 0.6	0.999
		20	234 ± 6	0.46 ± 0.11	0.922		
		40	262 ± 12	0.24 ± 0.06	0.916		
		52	366 ± 21	0.17 ± 0.05	0.903		
	S-0.25	8	129 ± 6	1.0 ± 0.3	0.915	−33.7 ± 0.9	0.999
		20	278 ± 6	0.52 ± 0.09	0.967		
		52	358 ± 22	0.14 ± 0.04	0.892		

**Table 5:** Phosphorus sorption values obtained in this work and reported in literature.

**Material**	**Q_o_ (mg∙g^−1^)**	**Reference**
GSP-Fe	366	This work
Commercial granular ferric hydroxide	105	[20]
La(OH)3 loaded magnetic mesoporous silica nanospheres	54.2	[23]
Double network CS/GA.20/PEI.15 cryogel	111.9	[25]
Sugarcane harvest Mg-biochar composite	399	[38]
Fe-impregnated waste activated sludge biochar	111.0	[39]
Hydrated iron oxides	111.1	[40]
Lanthanum-modified zeolites	61.1	[41]

## Data Availability

All data that support this work are included in this manuscript and in the Supplementary Materials.

## Supplementary Materials

The following supporting information can be downloaded at https://www.mdpi.com/article/10.3390/nano13071167/s1, Section: Models. Figure S1: Pore size distribution and cumulative pore volume of GSP S-1, GSP S-1 washed with deionised water to remove NaCl deposits, GSP-Fe S-1 and GSP-Fe S-1 with adsorbed phosphorus; Figure S2: SEM image and EDS maps of GSP S-1; Figure S3: SEM image and EDS spectrum of GSP-Fe S-1; Figure S4: SEM image and EDS maps of GSP-Fe S-0.25; Figure S5: (a) Experimental data of Fe sorption on GSP S-1, S-0.5 and S-0.25. (b) Experimental data of phosphorus sorption on GSP-Fe S-1, S-0.5 and S-0.25 referred to the iron. Solid lines were obtained using Equation (S5); Figure S6: (a) Experimental data of Fe sorption on GSP S-1, S-0.5 and S-0.25. (b) Experimental data of phosphorus sorption on GSP-Fe S-1, S-0.5 and S-0.25 refers to the iron. Solid lines were obtained using Equation (S7); Figure S7: (a) Experimental data obtained for phosphorus adsorption using GSP S-1. (b) Dependence of phosphorus adsorption with respect to the Fe present in the GSP-Fe; Figure S8: Effect of the ionic strength in phosphorus sorption by GSP-Fe S-1; Figure S9: (a) Influence of temperature on phosphorus adsorption using GSP-Fe S-0.25. (b) Representation of ln K vs. 1/T according to van ’t Hoff equation. Reference [42] is cited in the supplementary materials.

## References

[1] EuChemS The Periodic Table and Us. https://www.euchems.eu/the-periodic-table-and-us/.

[2] Emsley J. (2003). Nature’s Building Blocks: An A-Z Guide to the Elements.

[3] Martinez-Cabanas M., Lopez-Garcia M., Barriada J.L., Herrero R., Sastre de Vicente M.E. (2016). Green synthesis of iron oxide nanoparticles. Development of magnetic hybrid materials for efficient As(V) removal. Chem. Eng. J..

[4] E.U. (2020). Directive 2000/60/EC of the European Parliament and of the Council of 23 October 2000 Establishing a Framework for Community Action in the Field of Water Policy.

[5] U.S. E.P.A Nutrient Pollution: The Issue. https://www.epa.gov/nutrientpollution/issue.

[6] Azam H.M., Alam S.T., Hasan M., Yameogo D.D.S., Kannan A.D., Rahman A., Kwon M.J. (2019). Phosphorous in the environment: Characteristics with distribution and effects, removal mechanisms, treatment technologies, and factors affecting recovery as minerals in natural and engineered systems. J. Environ. Chem. Eng..

[7] E.U. (2010). Directive 2010/75/EU of the European Parliament and of the Council of 24 November 2010 on Industrial Emissions (Integrated Pollution Prevention and Control).

[8] European Environment Agency Industrial Reporting under the Industrial Emissions Directive 2010/75/EU and European Pollutant Release and Transfer Register Regulation (EC) No 166/2006. https://www.eea.europa.eu/data-and-maps/data/industrial-reporting-under-the-industrial-6.

[9] Dougherty W.J., Fleming N.K., Cox J.W., Chittleborough D.J. (2004). Phosphorus Transfer in Surface Runoff from Intensive Pasture Systems at Various Scales. J. Environ. Qual..

[10] Schoumans O.F., Chardon W.J., Bechmann M.E., Gascuel-Odoux C., Hofman G., Kronvang B., Rubæk G.H., Ulén B., Dorioz J.M. (2014). Mitigation options to reduce phosphorus losses from the agricultural sector and improve surface water quality: A review. Sci. Total Environ..

[11] Pavlidis G., Tsihrintzis V.A. (2018). Environmental Benefits and Control of Pollution to Surface Water and Groundwater by Agroforestry Systems: A Review. Water Resour..

[12] European Environment Agency Total Phosphorus in Lakes. https://www.eea.europa.eu/data-and-maps/daviz/total-phosphorus-in-lakes-2#tab-chart_1.

[13] European Environment Agency Phosphate in Rivers. https://www.eea.europa.eu/data-and-maps/daviz/phosphate-in-rivers-2/#tab-chart_1.

[14] Bacelo H., Pintor A.M.A., Santos S.C.R., Boaventura R.A.R., Botelho C.M.S. (2020). Performance and prospects of different adsorbents for phosphorus uptake and recovery from water. Chem. Eng. J..

[15] Muhmood A., Lu J., Dong R., Wu S. (2019). Formation of struvite from agricultural wastewaters and its reuse on farmlands: Status and hindrances to closing the nutrient loop. J. Environ. Manag..

[16] Suzuki K., Waki M., Yasuda T., Fukumoto Y., Kuroda K., Sakai T., Suzuki N., Suzuki R., Matsuba K. (2010). Distribution of phosphorus, copper and zinc in activated sludge treatment process of swine wastewater. Bioresour. Technol..

[17] Bustillo-Lecompte C.F., Mehrvar M. (2015). Slaughterhouse wastewater characteristics, treatment, and management in the meat processing industry: A review on trends and advances. J. Environ. Manag..

[18] Grzmil B., Wronkowski J. (2006). Removal of phosphates and fluorides from industrial wastewater. Desalination.

[19] Battistoni P., Carniani E., Fratesi V., Balboni P., Tornabuoni P. (2006). Chemical−Physical Pretreatment of Phosphogypsum Leachate. Ind. Eng. Chem. Res..

[20] Delgadillo-Velasco L., Hernández-Montoya V., Rangel-Vázquez N.A., Cervantes F.J., Montes-Morán M.A., Moreno-Virgen M.d.R. (2018). Screening of commercial sorbents for the removal of phosphates from water and modeling by molecular simulation. J. Mol. Liq..

[21] Zhou Z., Lu Y., Zhan W., Guo L., Du Y., Zhang T.C., Du D. (2022). Four stage precipitation for efficient recovery of N, P, and F elements from leachate of waste phosphogypsum. Miner. Eng..

[22] Mayer B.K., Gerrity D., Rittmann B.E., Reisinger D., Brandt-Williams S. (2013). Innovative Strategies to Achieve Low Total Phosphorus Concentrations in High Water Flows. Crit. Rev. Environ. Sci. Technol..

[23] Chen L., Li Y., Sun Y., Chen Y., Qian J. (2019). La(OH)3 loaded magnetic mesoporous nanospheres with highly efficient phosphate removal properties and superior pH stability. Chem. Eng. J..

[24] Feng Y., Lu H., Liu Y., Xue L., Dionysiou D.D., Yang L., Xing B. (2017). Nano-cerium oxide functionalized biochar for phosphate retention: Preparation, optimization and rice paddy application. Chemosphere.

[25] Dragan E.S., Humelnicu D., Dinu M.V. (2019). Development of chitosan-poly(ethyleneimine) based double network cryogels and their application as superadsorbents for phosphate. Carbohydr. Polym..

[26] Rice E.W., Baird R.B., Eaton A.D., Clesceri L.D. (2012). Standard Methods For the Examination of Water and Wastewater.

[27] Rice E.W., Baird R.B., Eaton A.D., Clesceri L.D. (2012). Standard Methods For the Examination of Water and Wastewater.

[28] Brunauer S., Deming L.S., Deming W.E., Teller E. (1940). On a Theory of the van der Waals Adsorption of Gases. J. Am. Chem. Soc..

[29] Naderi M., Tarleton S. (2015). Chapter Fourteen—Surface Area: Brunauer–Emmett–Teller (BET). Progress in Filtration and Separation.

[30] Schneider C.A., Rasband W.S., Eliceiri K.W. (2012). NIH Image to ImageJ: 25 years of image analysis. Nat. Methods.

[31] Gómez-Carnota D., Barriada J.L., Herrero R. (2022). Green development of iron doped silica gel materials for chromium decontamination. J. Environ. Chem. Eng..

[32] Boyd G.E., Adamson A.W., Myers L.S. (1947). The Exchange Adsorption of Ions from Aqueous Solutions by Organic Zeolites. II. Kinetics1. J. Am. Chem. Soc..

[33] Reichenberg D. (1953). Properties of Ion-Exchange Resins in Relation to their Structure. III. Kinetics of Exchange. J. Am. Chem. Soc..

[34] Kalavathy M.H., Karthikeyan T., Rajgopal S., Miranda L.R. (2005). Kinetic and isotherm studies of Cu(II) adsorption onto H3PO4-activated rubber wood sawdust. J. Colloid Interface Sci..

[35] Mohan D., Singh K.P. (2002). Single- and multi-component adsorption of cadmium and zinc using activated carbon derived from bagasse—An agricultural waste. Water Res..

[36] Sellaoui L., Guedidi H., Knani S., Reinert L., Duclaux L., Ben Lamine A. (2015). Application of statistical physics formalism to the modeling of adsorption isotherms of ibuprofen on activated carbon. Fluid Phase Equilib..

[37] Li Z., Sellaoui L., Dotto G.L., Bonilla-Petriciolet A., Ben Lamine A. (2019). Understanding the adsorption mechanism of phenol and 2-nitrophenol on a biopolymer-based biochar in single and binary systems via advanced modeling analysis. Chem. Eng. J..

[38] Li R., Wang J.J., Zhou B., Zhang Z., Liu S., Lei S., Xiao R. (2017). Simultaneous capture removal of phosphate, ammonium and organic substances by MgO impregnated biochar and its potential use in swine wastewater treatment. J. Clean. Prod..

[39] Yang Q., Wang X., Luo W., Sun J., Xu Q., Chen F., Zhao J., Wang S., Yao F., Wang D. (2018). Effectiveness and mechanisms of phosphate adsorption on iron-modified biochars derived from waste activated sludge. Bioresour. Technol..

[40] Acelas N.Y., Martin B.D., López D., Jefferson B. (2015). Selective removal of phosphate from wastewater using hydrated metal oxides dispersed within anionic exchange media. Chemosphere.

[41] Goscianska J., Ptaszkowska-Koniarz M., Frankowski M., Franus M., Panek R., Franus W. (2018). Removal of phosphate from water by lanthanum-modified zeolites obtained from fly ash. J. Colloid Interface Sci..

[42] Williams M., O’Neil M.J., Chemistry R.S.O. (2013). The Merck Index: An Encyclopedia of Chemicals, Drugs, and Biologicals.

