# Supplementary material for: Sustainable Low-Cost Phosphorus Recovery Using Nanostructured Materials with Reusability Potential
Source: Nanomaterials (Basel). 2023 Mar 24;13(7):1167. doi: 10.3390/nano13071167 (PMC10097266; doi:10.3390/nano13071167)
Supplement: Supplementary file 1 [file nanomaterials-13-01167-s001.zip › nanomaterials-2280361-supplementary.pdf]

Supplementary Materials

Sustainable Low-Cost Phosphorus Recovery Using Nanostructured Materials with Reusability Potential

David Gómez-Carnota, José L. Barriada *, Pilar Rodríguez-Barro, Manuel E. Sastre de Vicente and Roberto Herrero

Departamento de Química and CICA—Centro Interdisciplinar de Química e Bioloxía, Universidade da Coruña, As Carballeiras, s/n, 15071 A Coruña, Spain
* Correspondence: jose.barriada@udc.es; Tel.: +34-881012261

Models

Iron and phosphorus adsorption kinetics were studied using the Boyd diffusion model [1].

$$F = 1 - {}^{6}/_{\pi^2} \sum_{n=1}^{\infty} {}^{1}/_{n^2} \exp\left(- \frac{D_i \pi^2 n^2 t}{r^2}\right) \quad \text{(S1)}$$

where F is the fraction of metal removed at time t, and D_i is the effective diffusion coefficient of the sorbent in the solid phase ($cm^2 \cdot h^{-1}$) and r the radius of the solid particle (cm). Boyd encompasses the diffusion coefficient and the radius of the particle within a constant B defined as:

$$B = \frac{D_i \pi^2}{r^2} \quad \text{(S2)}$$

Boyd model can be applied in a simpler way. Reichenberg explains that at sufficiently high values of F, only one term of the series needs to be used [2]. Thus, Eq. (S1) is described through the expression:

$$Bt = -\ 0.4977 - \ln(1 - F) \quad \text{(S3)}$$

This expression provides small error values of Bt when F ≈ 1, but large error values at low F values. The error is maximised to −0.4977 when F = 0. To correct this fact, for the lower range of values of F (F < 0.86), Reichenberg proposes the transformation of Eq. (S3) to the following expression:

$$Bt = 2\pi - \frac{\pi^2 F}{3} - 2\pi \left(1 - \frac{\pi F}{3}\right)^{½} \quad \text{(S4)}$$

In the case of the equilibria studies, three isotherm models were used to describe the experimental data. The Langmuir and Langmuir-Freundlich models were chosen because they are two of the most widely used models to describe adsorption isotherms. Langmuir model is defined by the following expression:

$$q = Q_0 \frac{K \cdot c}{1 + K \cdot c} \quad \text{(S5)}$$

where q is the sorbent amount adsorbed at equilibrium, c is the concentration of sorbent in solution at equilibrium, Q0 is the maximum adsorption capacity and K is an affinity parameter.

The Langmuir-Freundlich model is described through the following equation:

$$q = Q_0 \frac{(K \cdot c)^{1/n}}{1 + (K \cdot c)^{1/n}} \tag{S6}$$

where n is a parameter related to surface heterogeneity and q, c, Q_0 and K are the same parameters as in the Langmuir equation.

Finally, the equilibria were studied using a model developed using statistical thermodynamics, described by Sellaoui et al. [3]. This model was chosen because of its versatility in studying adsorption mechanisms. In this case, in view of the experimental data and previous experience in anionic contaminant removal, the model for single adsorbate and monolayer adsorption was applied:

$$q = \frac{Q_0}{1 + (c_{½}/c)^m} \tag{S7}$$

where, $c_{½}$ is the sorbent concentration at half saturation, m is the number of atoms per adsorption site and q, c, Q_0 are the same parameters as in the previous equations.

Another reason to use this model is that it allows the calculation of the adsorption energy in a simple way through the following expression.

$$E = RT\ln {}^{c_s}/_{c_{½}} \tag{S8}$$

where cs is the solubility of the adsorbate, which has been obtained from bibliography [4].

The Van't Hoff equation was used in phosphorus adsorption studies at different temperatures to determine the standard enthalpy of reaction:

$$\frac{d \ln K}{dT} = \frac{\Delta H^{\circ}}{RT^2} \tag{S9}$$

Eq. (S9) can be integrated, assuming constant enthalpy:

$$\ln K = -\frac{\Delta H^{\circ}}{R} \cdot \frac{1}{T} + C \tag{S10}$$

By plotting ln K versus 1/T, if ΔH° remains essentially constant in the temperature range of the graph, a straight line is obtained. Thus, ΔH° can be calculated from the slope of a linear fit.

Figures

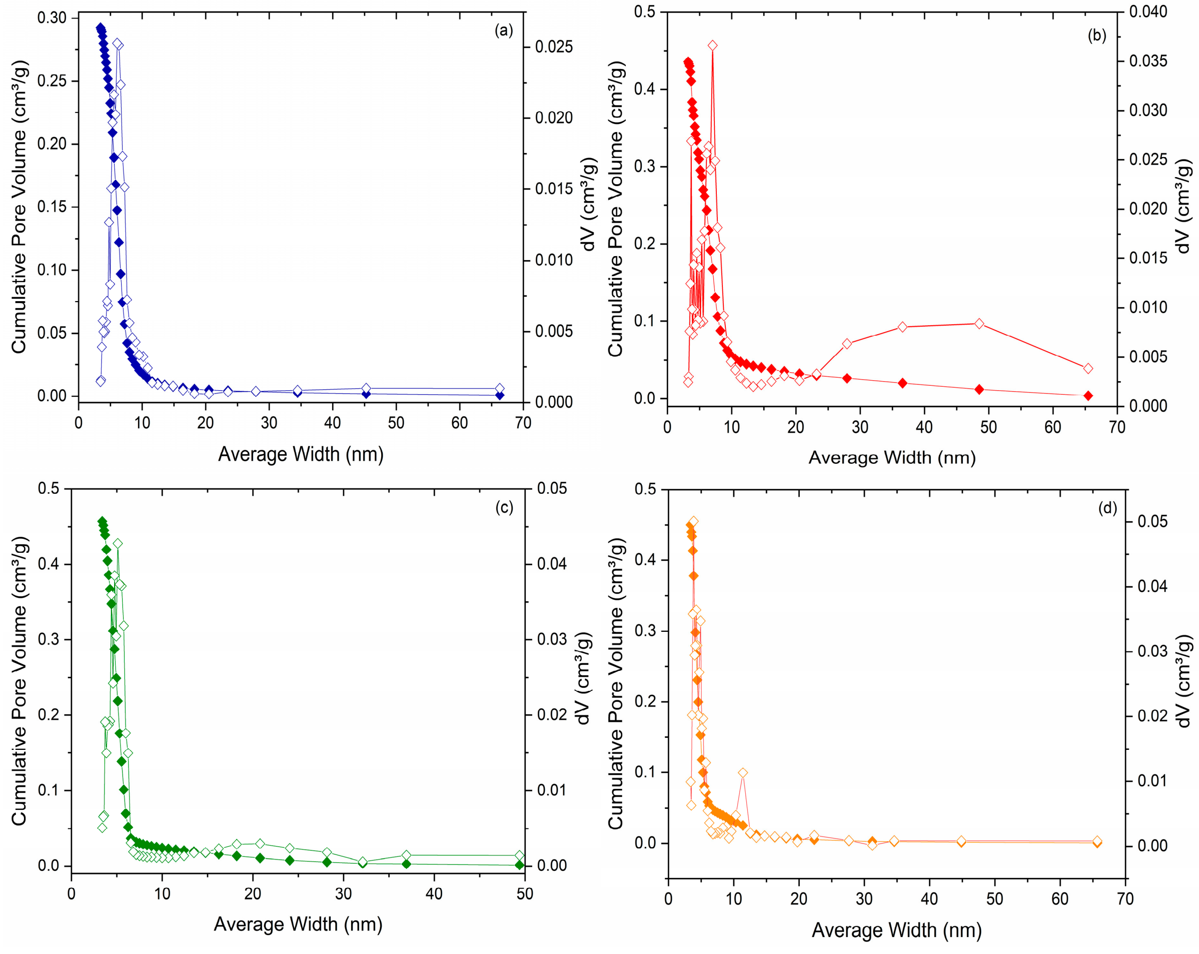

Figure S1. Pore size distribution (hollow diamonds) and cumulative pore volume (filled diamonds) of GSP S-1 (b, blue), GSP S-1 washed with deionised water to remove NaCl deposits (red), GSP-Fe S-1 (c, green) and GSP-Fe S-1 with adsorbed phosphorus (d, orange).

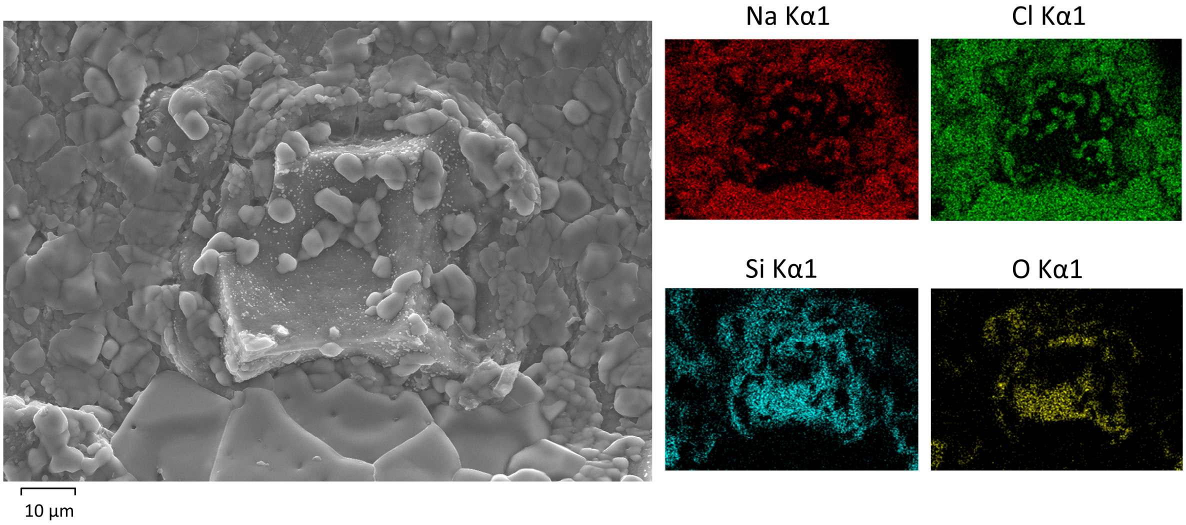

Figure S2: SEM image (1000x magnification) and EDS maps (Na, Red; Cl, green; Si, cyan; O, yellow) of GSP S-1.

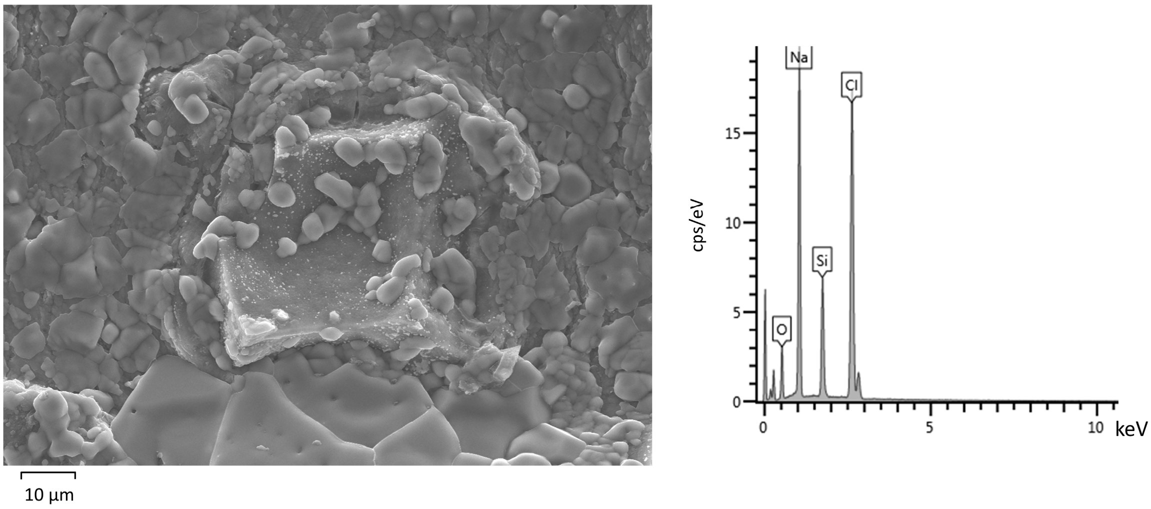

Figure S3: SEM image (1000x magnification) and EDS spectrum of GSP-Fe S-1

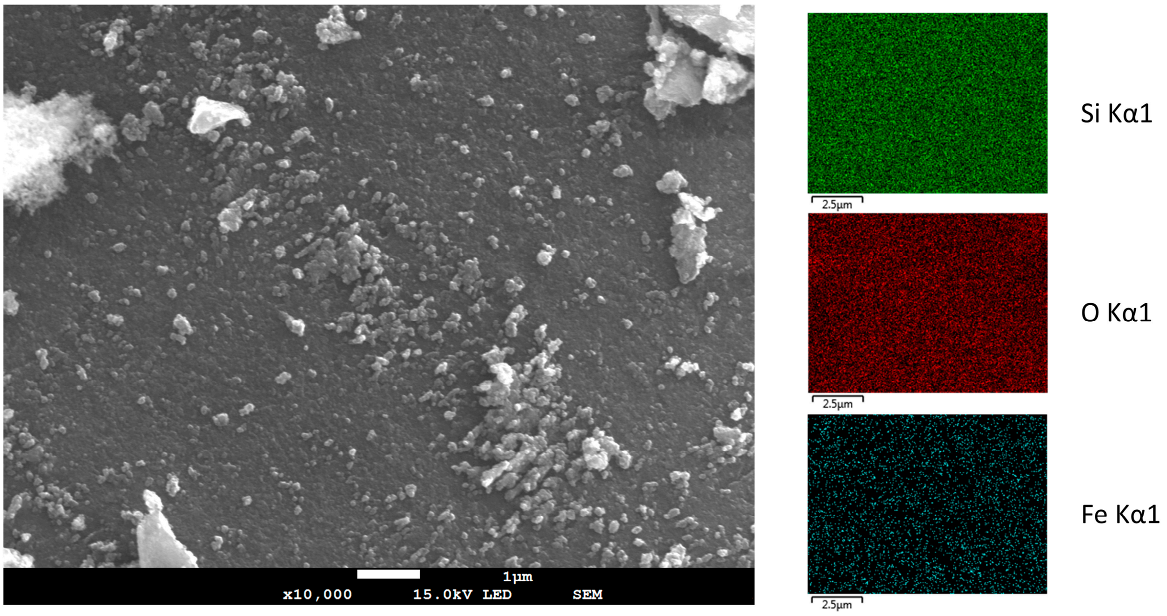

Figure S4. SEM image (10000x magnification) and EDS maps (Si, green; O, red; Fe, cyan) of GSP-Fe S-0.25.

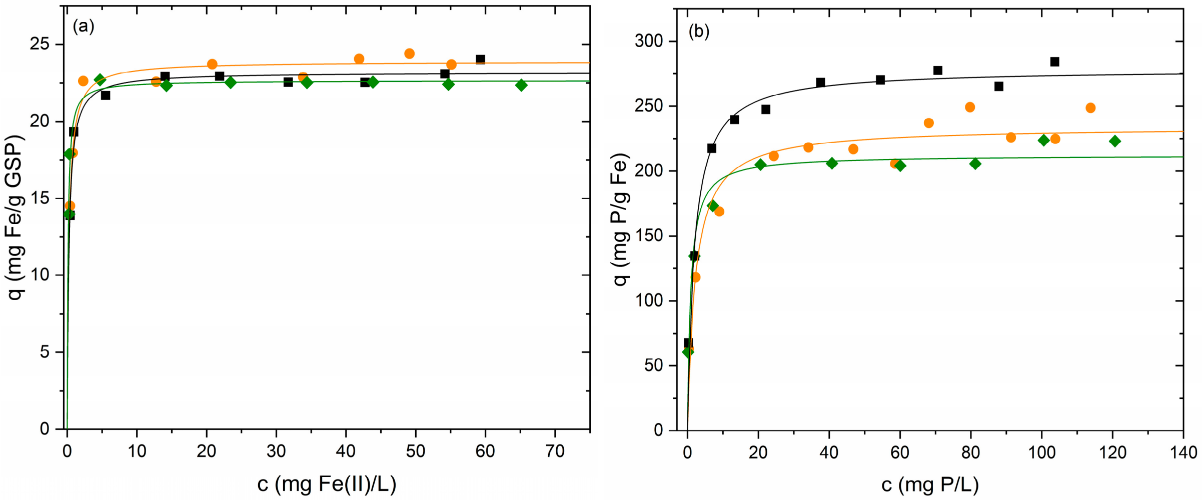

Figure S5. (a) Experimental data of Fe sorption on GSP (●) S-1, (■) S-0.5, (♦) S-0.25; Solid lines were obtained using Eq. (S5). GSP dose 2 g·L^{-1}, natural pH and room temperature. (b) Experimental data of phosphorus sorption on GSP-Fe referred to the iron. Solid lines were obtained using Eq. (S5). (●) GSP-Fe S-1. Dose 2 g·L^{-1}, which is equivalent to 47.4 mg Fe·L^{-1}. (♦) GSP-Fe S-0.5. Dose 2 g·L^{-1}, equivalent to 45.8 mg Fe ·L^{-1}. (■) GSP-Fe S-0.25. Dose 2 g·L^{-1}, equivalent to 45 mg Fe ·L^{-1}. pH 2.1 and room temperature for all phosphorus experiments.

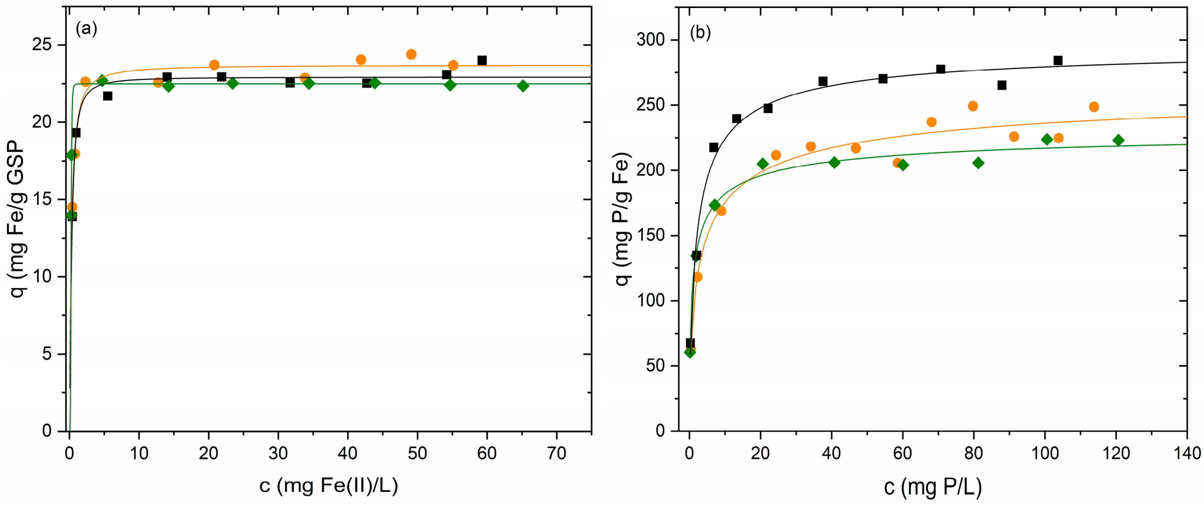

Figure S6. Experimental data of Fe sorption on GSP (●) S-1, (■) S-0.5, (♦) S-0.25; Solid lines were obtained using Eq. (S7). GSP dose 2 $g\cdot L^{-1}$, natural pH and room temperature. (b) Experimental data of phosphorus sorption on GSP-Fe referred to the iron. Solid lines were obtained using Eq. (S7). (●) GSP-Fe S-1. Dose 2 $g\cdot L^{-1}$, which is equivalent to 47.4 mg $Fe\cdot L^{-1}$. (♦) GSP-Fe S-0.5. Dose 2 $g\cdot L^{-1}$, equivalent to 45.8 mg $Fe\cdot L^{-1}$. (■) GSP-Fe S-0.25. Dose 2 $g\cdot L^{-1}$, equivalent to 45 mg $Fe\cdot L^{-1}$. pH 2.1 and room temperature for all phosphorus experiments.

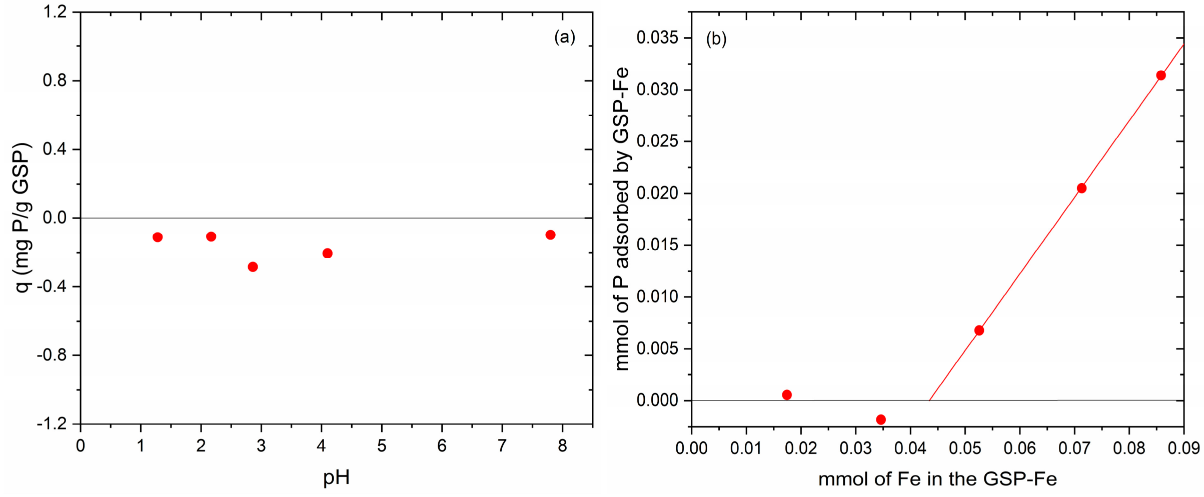

Figure S7. (a) Experimental data obtained for phosphorus adsorption using GSP S-1. Dose of GSP 2 $g\cdot L^{-1}$, phosphorus initial concentration 25 $mg\cdot L^{-1}$, room temperature and stirring at 175 RPM for all experiments. (b) Dependence of phosphorus adsorption with respect to the Fe present in the GSP-Fe. Lineal equation: $y = 740 \cdot 10^{-3} \pm 6 \cdot 10^{-3} x - 321 \cdot 10^{-4} \pm 5 \cdot 10^{-4}$.

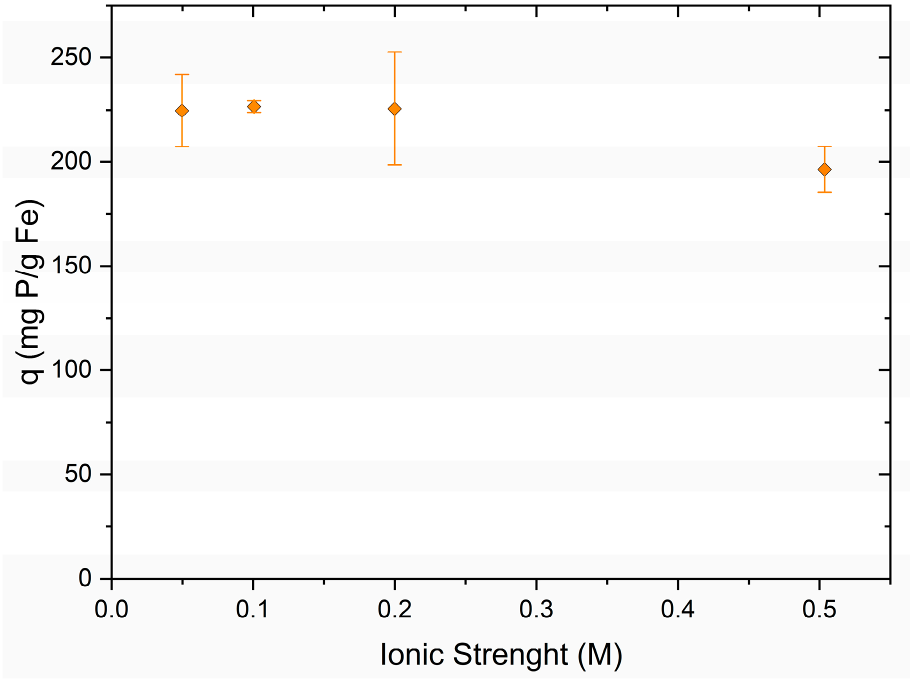

Figure S8. Effect of the ionic strength in phosphorus sorption by GSP-Fe S-1. GSP-Fe S-1 dose 2 g·L^{-1} equals to 47.4 mg Fe·L^{-1}, pH 2.1 and room temperature for all the experiments. Initial phosphorus concentration 80 mg·L^{-1}. Ionic strength was adjusted using KNO_3 as electrolyte.

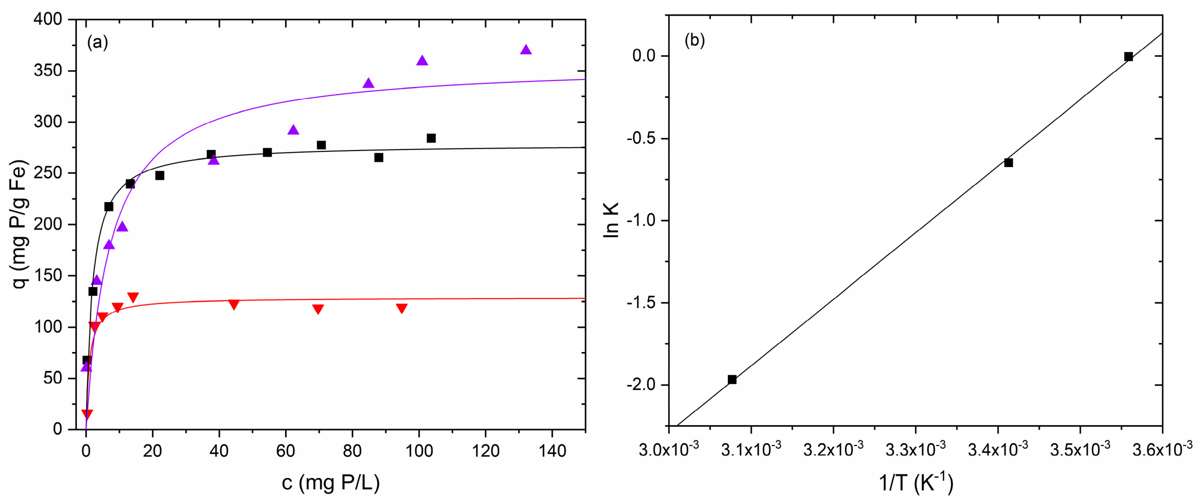

Figure S9. (a) Influence of temperature on phosphorus adsorption using GSP-Fe S-0.25. Dose 2 g·L^{-1}, equivalent to 45 mg Fe·L^{-1}. (▼) 8°C, (■) 20°C, , (▲) 52°C. pH 2.1 for all experiments. (b) Representation of ln K vs 1/T according to Van't Hoff equation.

References

1. Boyd, G.E.; Adamson, A.W.; Myers, L.S. The Exchange Adsorption of Ions from Aqueous Solutions by Organic Zeolites. II. Kinetics1. *J. Am. Chem. Soc.* **1947**, *69*, 2836-2848, doi:https://doi.org/10.1021/ja01203a066.
2. Reichenberg, D. Properties of Ion-Exchange Resins in Relation to their Structure. III. Kinetics of Exchange. *J. Am. Chem. Soc.* **1953**, *75*, 589-597, doi:https://doi.org/10.1021/ja01099a022.
3. Sellaoui, L.; Guedidi, H.; Knani, S.; Reinert, L.; Duclaux, L.; Ben Lamine, A. Application of statistical physics formalism to the modeling of adsorption isotherms of ibuprofen on activated carbon. *Fluid Phase Equilib.* **2015**, *387*, 103-110, doi:https://doi.org/10.1016/j.fluid.2014.12.018.

4. Williams, M. *The Merck Index: An Encyclopedia of Chemicals, Drugs, and Biologicals*, 15th ed.; O'Neil, M.J., Chemistry, R.S.o., Eds.; John Wiley & Sons, Ltd: Cambridge, UK, 2013; Volume 74, p. 2708.
